# Functions of NOD-Like Receptors in Human Diseases

**DOI:** 10.3389/fimmu.2013.00333

**Published:** 2013-10-16

**Authors:** Yifei Zhong, Anna Kinio, Maya Saleh

**Affiliations:** ^1^Department of Microbiology and Immunology, McGill University, Montreal, QC, Canada; ^2^Department of Medicine, McGill University, Montreal, QC, Canada

**Keywords:** NLR, inflammation, autoimmunity, IBD, polymorphisms, reproduction, innate immunity, infection

## Abstract

Nucleotide-binding and oligomerization domain NOD-like receptors (NLRs) are highly conserved cytosolic pattern recognition receptors that perform critical functions in surveying the intracellular environment for the presence of infection, noxious substances, and metabolic perturbations. Sensing of these danger signals by NLRs leads to their oligomerization into large macromolecular scaffolds and the rapid deployment of effector signaling cascades to restore homeostasis. While some NLRs operate by recruiting and activating inflammatory caspases into inflammasomes, others trigger inflammation via alternative routes including the nuclear factor-κB, mitogen-activated protein kinase, and regulatory factor pathways. The critical role of NLRs in development and physiology is demonstrated by their clear implications in human diseases. Mutations in the genes encoding NLRP3 or NLRP12 lead to hereditary periodic fever syndromes, while mutations in *CARD15* that encodes NOD2 are linked to Crohn’s disease or Blau’s syndrome. Genome-wide association studies (GWASs) have identified a number of risk alleles encompassing NLR genes in a host of diseases including allergic rhinitis, multiple sclerosis, inflammatory bowel disease, asthma, multi-bacillary leprosy, vitiligo, early-onset menopause, and bone density loss in elderly women. Animal models have allowed the characterization of underlying effector mechanisms in a number of cases. In this review, we highlight the functions of NLRs in health and disease and discuss how the characterization of their molecular mechanisms provides new insights into therapeutic strategies for the management of inflammatory pathologies.

## Introduction

The mammalian immune system encompasses an ancient genome-encoded innate immune system and a more recently acquired adaptive immune system capable of combating pathogens with exquisite specificity and long-term memory. The innate immune system remains a pivotal player in controlling host resistance. This system is equipped with an arsenal of pattern recognition receptors (PRRs) that translate microbial and danger sensing into immediate host defenses as well as provides signals to prime the adaptive immune response for long-lasting protection (1, 2). Nucleotide-binding and oligomerization domain (NOD)-like receptors (NLRs) are a group of evolutionarily conserved intracellular PRRs that play a vital role in innate immunity and host physiology, as reflected by their prevalence among living organisms of both the plant and animal kingdoms (3–9). In humans there are 22 known NLRs, and the association of mutations and single nucleotide polymorphisms (SNPs) in their genes with human diseases reflect their vital role in host defense. The function of NLRs is not restricted to immunity, as they also play important roles in reproduction and embryonic development (10–12).

**Figure 1:**
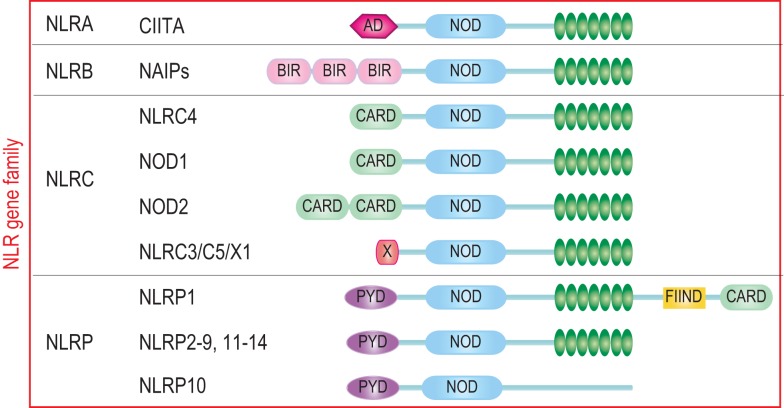
**The human NLR gene family**. The human NLR gene family consists of 22 members that share a tripartite structure, consisting of an N-terminal signaling domain, a central nucleotide-binding and oligomerization domain, and a C-terminal agonist sensing/ligand-binding domain. The NLR family is sub-divided into four sub-groups NLRA, NLRB, NLRC, and NLRP based on the nature of the N-terminal domain consisting respectively of an acidic transactivation domain, a baculovirus IAP repeat (BIR), a caspase-recruitment and activation domain (CARD), and a Pyrin domain (PYD).

**Figure 2:**
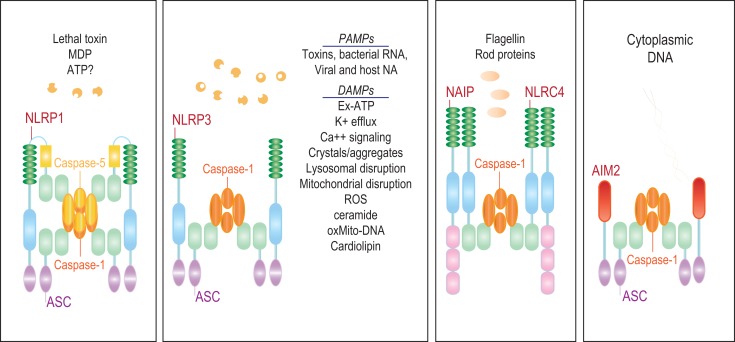
**The NLR inflammasomes**. The three biochemically characterized inflammasomes are depicted. The NLRP1 inflammasome consists of NLRP1, ASC, and caspases-1 and -5. Little is known about the agonists that activate NLRP1. Anthrax lethal toxin, MDP, and decreased cytosolic ATP have been reported to stimulate this inflammasome. NAIP and NLRC4 form a caspase-1 inflammasome in response to bacterial flagellin and T3SS rod proteins. NLRP3, on the other hand, is activated by a wide range of agonists including a number of MAMPs and DAMPs.

**Figure 3:**
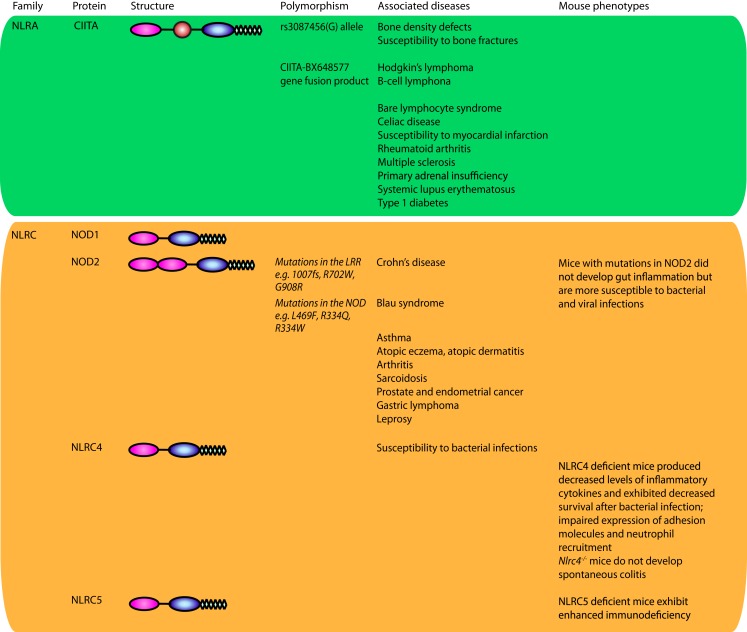

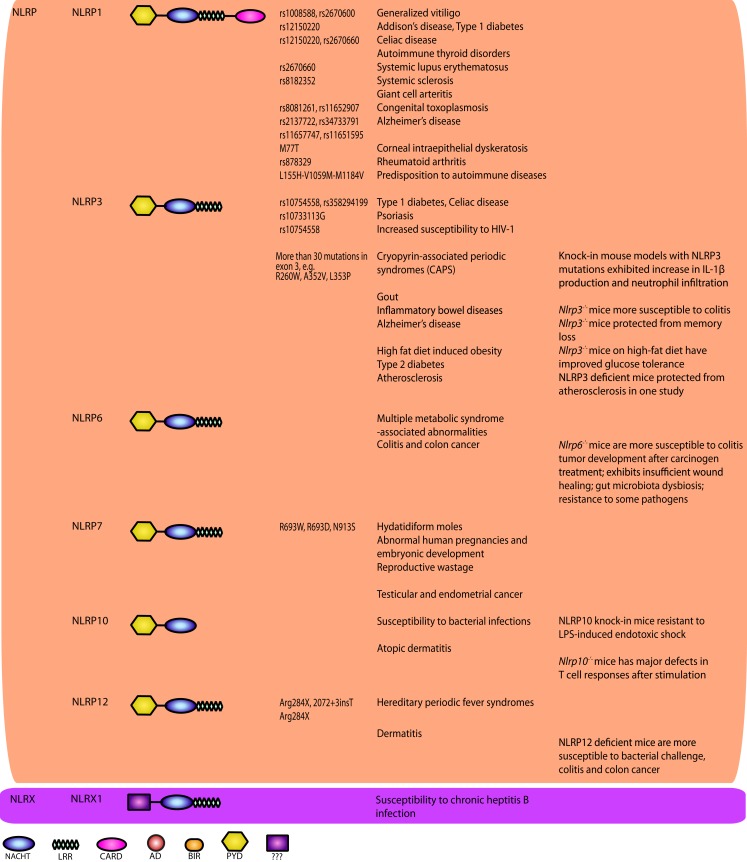
**NOD-like receptors and disease**. NLRs have been implicated in a plethora of diseases. Genetic studies have uncovered a number of variants in genes encoding NLRs or their signaling mediators associated with human diseases. Animal models have served as a key discovery platform to characterize the underlying functions and molecular mechanisms of NLRs in these diseases and associated pathologies. Together, these efforts have led to therapeutic success in the clinic for a subset of NLR-dependent auto-inflammatory diseases. When available, the mutations and SNPs linked to disease are listed and animal phenotypes are presented.

The characteristic feature of NLRs is a central NOD (or NACHT) domain, required for oligomerization, an N-terminal homotypic protein–protein interaction domain and a C-terminal series of leucine-rich repeats (LRRs) involved in agonist sensing or ligand binding. Mammalian NLRs are sub-divided into four subfamilies based on the variation in their N-terminal domain: NLRA or Class II transactivator (CIITA) contains an acid transactivation domain, NLRBs or neuronal apoptosis inhibitor proteins (NAIPs) possess a baculovirus inhibitor of apoptosis protein repeat (BIR), NLRCs have a caspase-recruitment domain (CARD), and NLRPs a pyrin domain (PYD). NLRX1 contains a CARD-related X effector domain (Figure 1). Upon ligand binding, the auto-inhibitory LRR undergoes a conformational change, which exposes the N-terminal domain allowing interaction with downstream signaling adaptors or effectors and formation of an oligomeric complex (13, 14). NLR platforms that recruit and activate the inflammatory protease caspase-1 are referred to as inflammasomes. Caspase-1 is required for the processing and maturation of inflammatory cytokines IL-1β and IL-18 and the induction of an inflammatory form of cell death termed pyroptosis (15, 16). Among the NLRs, NLRP1, NLRP3, NLRP6, NLRP7, NLRP12, NLRC4, and NAIP have been reported to operate via inflammasomes (Figure 2). Other NLRs such as NOD1, NOD2, NLRP10, NLRX1, NLRC5, and CIITA do not directly engage the inflammatory caspases, but instead activate nuclear factor-κB (NF-κB), mitogen-activated protein kinases (MAPKs), and interferon (IFN) regulatory factors (IRFs) to stimulate innate immunity. Below, we discuss the different NLRs along with their mechanisms of activation and diseases associated with defects in their activities (Figure 3).

## Inflammasome-Forming NLRs

In 2001, the causative mutation of Muckle–Wells Syndrome (MWS), a rare autosomal recessive auto-inflammatory disease, was mapped to *NLRP3* (*CIAS1*) (17). In 2002, Tschopp and colleagues were the first to characterize the inflammasome, defining it biochemically as a complex consisting of an NLR (NLRP1), the bipartite adaptor protein ASC (which contains both a CARD and a PYD), and the two inflammatory caspases, caspases-1 and -5 (18). In 2004, the discovery of the links between the *NLRP3* mutations, NLRP3 inflammasome hyper-activation, and excessive production of IL-1β has set the stage for the use of IL-1 blockade strategies, such as recombinant IL-1 receptor antagonist (anakinra) or anti-IL-1β antibodies (canakinumab), to cure patients inflicted with hereditary periodic fever syndromes [reviewed in Ref. (19)]. Concurrently, Dixit and colleagues reported the generation of the first inflammasome knockouts, namely mice deficient in IPAF (NLRC4) or the adaptor ASC, and showed that macrophages from these mice had a defect in IL-1β production following infection with flagellated bacteria (20). As more inflammasome-forming NLRs are continuously being characterized and studied, their importance in activating immune responses and consequently in conferring host resistance is becoming evident.

### NLRP1

The NLRP1 protein has a unique structure amongst other NLRs. Human NLRP1 contains a PYD on the N-terminus and a CARD on the C-terminus, with ZU5 and UPA domains in the internal region which confers proteolytic activity upon the protein (21). Three murine NLRP1 homologs – Nlrp1a, Nlrp1b, and Nlrp1c – have been identified, although they lack the N-terminal PYD domain present in human NLRP1. Few ligands have been found for NLRP1 to date, and include bacterial products such as lethal toxin (LT) produced by *Bacillus anthracis* which activates murine NLRP1b (22), muramyl dipeptide (MDP), a component of bacterial peptidoglycan that activates human NLRP1; and reduced levels of cytosolic ATP (23–27).

Defects in NLRP1 have been linked to a variety of autoimmune disorders. Candidate gene analysis and Genome-wide association studies (GWAS) have shown a significant association of polymorphic variants in the extended promoter and/or coding regions of *NLRP1* with familial cases of generalized vitiligo (28, 29), celiac disease (30), Addison’s disease and type 1 diabetes (31, 32), autoimmune thyroid disorders (AITDs) (33), systemic lupus erythematosus (SLE) (34), systemic sclerosis and giant cell arteritis (35, 36), congenital toxoplasmosis (37), rheumatoid arthritis (38), and Alzheimer’s disease (39) (Figure 3). A novel missense mutation M77T in *NLRP1*, which destabilizes the protein, has been recently shown to cause corneal intraepithelial dyskeratosis (40).

In a recent analysis of high-risk haplotypes with substitutions in human *NLRP1*, Levandowski et al. demonstrated that peripheral blood monocytes from heterozygous carriers of the haplotype 2A (which contains three non-synonymous substitutions: L155H-V1059M-M1184V) process significantly greater amounts of pro-IL-1β into mature IL-1β under basal conditions. It was thus proposed that the enhanced production of IL-1β predisposes carriers to a wide spectrum of autoimmune diseases (41). Consistently, patients diagnosed with vitiligo commonly suffer from other autoimmune disorders such as SLE (28, 42). However, the molecular mechanisms underlying the link between *NLRP1* genetic variations and these disorders are still unknown. It is plausible that deregulation of an NLRP1 inflammasome effector function is at the basis of the autoimmunity phenotypes. This is consistent with recent results from mice. Masters et al. have recently reported that mice with an activating mutation in *Nlrp1a* exhibited increased T-cell progenitor death (pyroptosis) at the steady state, which rendered them cytopenic (43). In contrast, *Nlrp1a*-deficient mice, which may experience less pyroptosis, develop an over-exuberant immune response (43). However, while Masters et al. demonstrated that the inflammatory disease in *Nlrp1a* mutant mice was dependent on caspase-1, additional proof is needed to show that Nlrp1a formed an inflammasome complex *in vivo* (43). While anakinra has been shown to be successful in treating patients with SLE in preliminary studies, IL-1 blockade strategies have not been tested to date for other autoimmune diseases such as vitiligo or celiac disease (42, 44).

### NLRP3

The NLRP3 inflammasome is arguably the most studied inflammasome to date. NLRP3 is predominantly expressed in splenic neutrophils, macrophages, monocytes, and conventional dendritic cells, and its expression is inducible in response to inflammatory stimuli (45). There is evidence suggesting that a two-step process is required for NLRP3 activation. The first, or priming signal, converges on the activation of NF-κB and transcriptional induction of inflammasome components including NLRP3 itself and pro-IL-1β. The second, or activating signal, in the form of a microbial or danger signal, is then able to directly activate inflammasome assembly (46). NLRP3 is able to recognize a wide variety of exogenous and endogenous stimuli such as microbial agonists, ATP, and particulate matters (47, 48). There is, however, scarce evidence that NLRP3 binds directly to its activators. Instead its activation is thought to be triggered by signaling intermediates (46). For instance, Shenoy et al. proposed that guanylate binding protein 5 (GBP5) may play a vital role in activating inflammasome assembly and promoting caspase-1 processing in response to live bacteria and bacterial cell wall components (49). A recent study by Zhong et al. suggested that particulate stimuli might induce mitochondrial production of reactive oxygen species (ROS), which triggers a calcium influx mediated by transient receptor potential melastatin 2 (TRPM2) to activate NLRP3 (50). The role of ROS in NLRP3 activation is consistent with earlier results by Zhou et al. who showed that ROS also leads to the dissociation of thioredoxin-interacting protein (TXNIP) from thioredoxin, freeing it to interact with and activate NLRP3 (51). In addition, it has been reported that NLRP3 activators are able to disrupt the mitochondria, resulting in the release of oxidized mitochondrial DNA and/or cardiolipin, which can bind to and activate NLRP3 (52, 53). Alternatively, it was argued that mitochondrial disruption is not required for NLRP3 activation; instead K^+^ efflux is sufficient to stimulate this NLR (54). NLRP3 inflammasome activation may involve at least two adaptors, ASC and the mitochondria-associated adaptor MAVS. It was recently shown that MAVS recruits NLRP3 to the mitochondria for activation in response to non-crystalline activators (55) and that microtubule-driven trafficking of the mitochondria is necessary for NLRP3-ASC complex assembly and activation (56).

Gain-of-function mutations in the *NLRP3* gene were first associated with cryopyrin-associated periodic fever syndromes (CAPS), which are a group of rare hereditary auto-inflammatory diseases including familial cold urticaria, MWS, and neonatal onset multisystem inflammatory disease [reviewed in Ref. (19)]. Mutations in *NLRP3* were reported to induce an overproduction of IL-1β that triggers the subsequent development of severe inflammation (57, 58). A knock-in mouse model of MWS have validated the observations made in human patients, and showed that equivalent mutations in murine *Nlrp3* lead to the production of massive amounts of IL-1β, which mediates the disease (59, 60). IL-1 blockade therapies are frequently used to treat auto-inflammatory diseases. Anakinra and canakinumab, for example, have been used to treat CAPS patients with great success, as several groups have reported long-lasting clinical responses as well as the restoration of IL-1β production levels to normal amounts in patients after treatment (61, 62).

NLRP3 was also linked to gout, which is a result of uric acid crystal deposition in the joints as a consequence of a rich diet high in purines (63). The exact mechanism of NLRP3 activation by uric acid crystals is still unknown, but monosodium urate and calcium pyrophosphate dihydrate crystals were found to induce NLRP3 and caspase-1 activation and the subsequent processing of IL-1β and IL-18 (64). Since uric acid can also be released from dying cells as a DAMP (65), there has been speculation that NLRP3 may also detect danger signals released from dying cells (66).

Single nucleotide polymorphisms in the NLRP3 locus have been associated with a wide range of disorders, including type 1 diabetes (67), celiac disease (67), psoriasis (68), and increased susceptibility to HIV-1 infections (69). While no SNP in the NLRP3 region is directly associated with inflammatory bowel disease (IBD), a SNP downstream of *NLRP3* has been previously identified as a risk allele in Crohn’s disease (70). Lewis and colleagues, however, were unable to reproduce this result, as they found no significant association between *NLRP3* SNPs and Crohn’s disease (71). A recent GWAS meta-analysis has shown that SNPs that affect receptors downstream of NLRP3 such as IL18R1, IL1R1, IL1RL1, IL1RL2, and IL1R2, are associated with susceptibility to IBD (72). Thus, although there is conflicting data regarding the effects of *NLRP3* variants in IBD, defects in inflammasome signaling likely play a role in IBD pathogenesis. Consistently, *Nlrp3*^−/−^ mice or mice deficient in inflammasome components were found to be significantly more susceptible to experimental models of colitis compared to wild-type mice (73–76). Together, these studies indicate that NLRP3 may be involved in intestinal tissue repair mechanisms following injury.

The NLRP3 inflammasome has also been implicated in different metabolic pathologies. For instance, the NLRP3 inflammasome has been linked to obesity, insulin resistance, atherosclerosis, and Alzheimer’s disease. It has been shown that activation of caspase-1 and IL-1β processing downstream of NLRP3 lead to inhibition of adipocyte differentiation and contributes to high fat diet-induced obesity (77). Several studies have also shown that the NLRP3 inflammasome may play a crucial role in insulin resistance and the potential development of type 2 diabetes (51, 77, 78). Consistently, *Nlrp3*^−/−^ or *Asc*^−/−^ mice were reported to have improved glucose tolerance and insulin sensitivity when fed a high fat diet. Ceramide, a specific product from the metabolism of long-chain saturated fatty acids, and the saturated free fatty acid, palmitate, have been shown to induce IL-1β in an NLRP3-dependent fashion [Ref. (78) and reviewed in Ref. (63)]. IL-1β produced downstream of the NLRP3 inflammasome, which is also stimulated by islet amyloid polypeptide (79), promotes beta-cell dysfunction, and cell death (80), linking NLRP3 activation to insulin resistance. Crystalline cholesterol was proposed to cause atherosclerosis by acting as a danger signal and initiating inflammation through the NLRP3 inflammasome. Consistently, Duewell et al. observed that mice deficient in components of the NLRP3 inflammasome did not undergo acute inflammation after the injection of cholesterol crystals, and had markedly decreased atherosclerosis compared to wild-type animals (81). There is, however, some controversy in this area, as Menu et al. reported no differences in disease progression in *Nlrp3*^−^*^/^*^−^mice compared to wild-type animals (82). In Alzheimer’s disease, amyloid-β aggregates were shown to activate NLRP3 *ex vivo* in primary macrophages and microglia (83). This was supported by *in vivo* results by Heneka et al. who demonstrated that NLRP3 deficiency protected mice with familial Alzheimer’s disease mutations from memory loss (84).

### NLRP6

Preliminary immunofluorescence data has proposed that the formation of the NLRP6 inflammasome is dependent on the recruitment of NLRP6 to ASC specks in the cytosol via its N-terminal PYD (85, 86). While much of its functions still remain unknown, recent studies have demonstrated that NLRP6 is important in the self-renewal and integrity of the intestinal epithelium, as *Nlrp6*^−^*^/^*^−^ mice exhibited insufficient wound healing after injury (87, 88) and were more susceptible to carcinogen-induced tumor development compared with wild-type mice (89, 90). While the precise mechanisms by which NLRP6 protects against tumorigenesis is not clear. It is known that *Nlrp6*^−^*^/^*^−^ mice are able to sustain increases in intestinal epithelial proliferative activity over longer periods of time along with the observed lower efficiency in wound repair – in other words, the repair mechanism in *Nlrp6*^−^*^/^*^−^mice fails to promote wound healing but is still able to promote general cell proliferation, leading to higher incidents of dysplasia and tumorigenesis (91).

In a study by Elinav et al., *Nlrp6*^−^*^/^*^−^ mice showed an altered gut microbiota with an increase in colitogenic bacterial strains such as *Prevotellaceae* and TM7, which are also found in increased numbers in IBD patients, indicating a role for NLRP6 in the regulatory sensing system in the gut as well (92). The authors speculated that NLRP6 may act as a “gate keeper” by sensing bacterial products or cell damage and promoting the production of IL-18 during homeostasis which in turn supports the normal microbial flora in the gut to prevent dysbiosis (92). While this proposal raised some interesting points as to the functions of NLRP6 in the gut, a conclusion cannot be drawn as to whether the altered microbiota in *Nlrp6*^−^*^/^*^−^ animals is due to the absence of the protein, as no littermate analysis or maternal microbiota transfer experiments were conducted by the authors to further their hypothesis. A study by Henao-Mejia et al. later linked changes in *Nlrp6*^−^*^/^*^−^ mice microbiota to metabolic diseases (93). Namely, NLRP6 seems to negatively regulate the progression from non-alcoholic fatty liver disease to non-alcoholic steatohepatitis by preventing the increase in colitogenic bacteria (93). Additionally, Anand et al. observed that *Nlrp6*^−^*^/^*^−^ mice were highly resistant to a variety of pathogens including *Listeria monocytogenes* and *Escherichia coli* (94). *Nlrp6*^−^*^/^*^−^mice had increased numbers of immune cells in their circulation, as well as enhanced activation of MAPK and NF-κB signaling, though Toll-like receptor (TLR) activation, suggesting that NLRP6 may suppress TLR pathways after the recognition of pathogens to prevent amplified inflammatory pathology (94). The exact mechanisms of how NLRP6 functions, however, still remain to be studied.

### NLRP7

NLRP7, a human NLR with no murine orthologs, is characterized by an N-terminal PYD along with a NACHT domain and a C-terminal LRR region. Mutations in the *NLRP7* gene are associated with recurrent hydatidiform moles and reproductive wastage (substitutions R693W, R693P, and N913S) (11, 95–97). Furthermore, NLRP7 expression is increased in certain type of cancers such as testicular (98) and endometrial (99) cancers. However, the mechanisms underlying these phenotypes are not clear. Messaed et al. showed that PBMCs from patients with *NLRP7* mutations (at G118X, G380R, C399Y, R693W, A719V) secreted significantly lower levels of IL-1β and TNF in response to LPS despite high intracellular levels of pro-IL-1β and unimpaired pro-IL-1β processing. The authors concluded that NLRP7 might play a role in cytokine trafficking and secretion from the cell (100). Conversely, others have shown that overexpression of NLRP7 inhibited pro-IL-1β synthesis and secretion (88, 101). Moreover, it was recently reported that bacterial acylated lipopeptides (acLP) activated NLRP7 and stimulated formation of an NLRP7-ASC-caspase-1 inflammasome (102). Thus, further studies are needed to clarify NLRP7 mechanisms of actions and functions in reproduction and immunity.

### NLRP12

NLRP12 was previously reported to form an inflammasome as well as function in modulating NF-κB signaling (see below). A recent study by Vladimer et al. has shown that the NLRP12 inflammasome has a key role in controlling IL-1β and IL-18 production after *Yersinia pestis* infection, where NLRP12-deficient mice were more susceptible to infection compared to the controls (103). Other pathogens such as *Klebsiella pneumoniae* and *Mycobacterium tuberculosis* and bacterial components such as LPS do not seem to depend on NLRP12 for infection or pathology (104).

While many of the functions and activators of NLRP12 remain unknown, mutations in the NLRP12 gene have been associated with auto-inflammatory diseases such as atopic dermatitis (105) and hereditary periodic fever syndromes (106, 107). Anti-IL-1 therapies, similar to those administered to patients with NLRP3 mutations, have been conducted on patients with NLRP12 mutations with limited success. Patients treated with anakinra showed improvements early on during the treatment process, but developed resistance to the drug within months and suffered from severe myalgia as a side effect (107). Similarly, levels of IL-1β in these patients returned to pre-treatment levels after 14 months of treatment (107). Further clinical studies are needed before conclusions are drawn regarding the efficacy of anti-IL-1 agents in the treatment of diseases associated with NLRP12.

There is currently much debate as to the role of NLRP12 in inflammation, and both stimulatory and inhibitory functions have been proposed. Some studies have suggested that NLRP12 may negatively regulate the NF-κB pathway (86, 108, 109). *Nlrp12*^−^*^/^*^−^mice were found to be more susceptible to colitis and colon cancer, and polyps isolated from these mice showed significantly higher non-canonical activation of NF-κB with an increased expression of inflammation and cancer-related genes (109, 110). Conversely, Arthur et al. demonstrated in a murine model of allergic dermatitis that proinflammatory cytokine production was unaffected in *Nlrp12*^−^*^/^*^−^mice (111). Instead, dendritic cells in *Nlrp12*^−^*^/^*^−^ mice exhibited a much-reduced migratory capacity, and neither peripheral dendritic cells nor neutrophils in *Nlrp12*^−^*^/^*^−^mice responded to chemotaxic signals or chemokines in *in vitro* experiments (111). Yet another function for NLRP12 was proposed by Jéru et al., who discovered that mutations in *NLRP12* did not affect NF-κB activation, but rather increased ASC speck formation and caspase-1 activation (112). Altogether, these results suggest that NLRP12 plays a role in suppressing NF-κB while stimulating the inflammasome and assisting in the migration of immune cells.

### NLRC4 and NAIPs

NLRC4 possesses an N-terminal CARD that allows direct interaction with caspase-1 independently of ASC (113, 114). A recent study by Qu et al., showed that the phosphorylation of Ser533 in NLRC4 by PCKδ was crucial for the activation of the NLRC4 inflammasome (115). NAIPs, members of the NLRB sub-family, have been identified as critical components of the NLRC4 inflammasome. They are required for the recognition of bacterial components, as well as the scaffolding of the NAIP-NLRC4 inflammasome. Activators of this inflammasome include bacterial flagellin and components of the bacterial type III secretion system (T3SS) (113, 116, 117). Notably, murine Naip5 and Naip6 were shown to recognize bacterial flagellin and subsequently bind to NLRC4 to trigger the formation of the inflammasome, whereas Naip2 and human NAIP serve as receptors for the rod and the needle components, of the bacterial T3SS (118, 119).

NLRC4 plays an essential role in host survival and pathogen clearance following host infection with pathogens such as *Legionella pneumophila* (120, 121), *Candida albicans* (122), and *Burkholderia pseudomallei* (123). More recently, Cai et al. showed that, upon *K. pneumoniae* infections, *Nlrc4*^−^*^/^*^−^mice exhibited decreased survival compared to wild-type animals (124). Similarly, Franchi et al. reported that *Nlrc4*^−^*^/^*^−^ mice were highly susceptible to orogastric *Salmonella* infections (125). Interestingly, *Nlrc4*^−^*^/^*^−^mice do not develop spontaneous colitis in response to the commensal microbiota (126), likely due to low soluble flagellin levels in the gut and a primary role of TLR5 in dealing with gut flagellated bacteria. NLRC4 thus serves as an additional sentinel against pathogenic enteric infections (126, 127). NLRC4 has been previously been shown to act in sync with NLRP3 during *Salmonella* infection (114). More recently, it was demonstrated that both NLRs play non-redundant roles in *B. pseudomallei* infection and melioidosis, where NLRC4 is critical for pyroptosis and NLRP3 for the production of IL-1β and IL-18 (123). Ceballos-Olvera et al. demonstrated that while IL-18 and pyroptosis are both essential for host resistance, the production of IL-1β by NLRP3 was deleterious, as it triggered excessive neutrophil recruitment and exacerbated the disease (123). Thus NLRC4 seems to act synergistically with both TLR5 and NLRP3, but its contributions to their functions seems to be secondary.

## Non-Inflammasome-Forming NLRs

### NOD1/2

**Figure 4:**
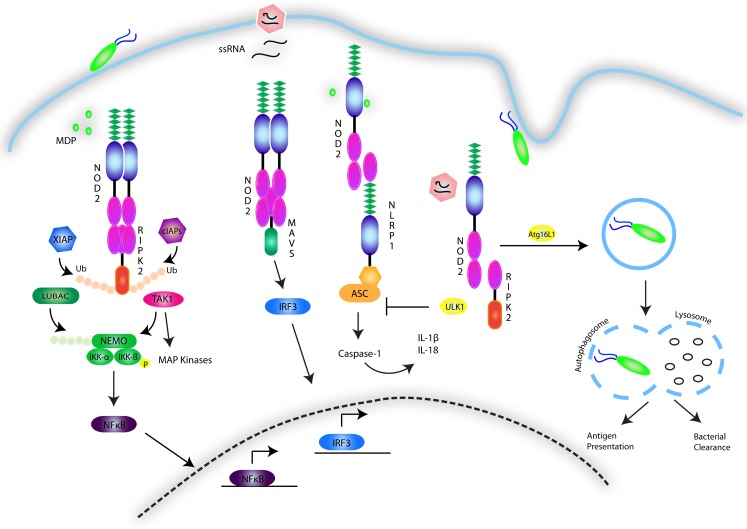
**NOD1/2 signaling pathways**. The NOD1 and 2 receptors recognize the bacterial peptidoglycan derivatives DAP and MDP. The events involved in signal transduction are depicted and involve the formation of a nodosome complex that is stabilized through a series of ubiquitin scaffolds mediated by a number of E3 ligases including cIAP1/2, XIAP, LUBAC, and ITCH. These scaffolds serve to engage effector kinases, including TAK1 and the IKK complex to activate NF-κB and MAPK pathways. NOD2 is additionally activated by single-stranded RNA viruses and stimulates an antiviral innate immune response by engaging MAVS and activating IRF3. The NOD receptors have also been shown to synergize with NLRP sensors to activate the inflammasome. Conversely, they have also been implicated in triggering autophagy though association with ATG16L1, and in response to viral infection, to inhibit the inflammasome by upregulating ULK1-dependent mitophagy.

NOD1 and 2, have been studied primarily in the context of their signaling activity following recognition of the peptidoglycan components diaminopimelic acid (DAP) and MDP from Gram-negative and Gram-positive bacteria (128–132). Despite this focus, much of the nature of the NOD1 and 2 interaction with these structures remains unknown, although recent findings suggest that NOD2 directly binds MDP with high affinity (133), with the N-glycosylated form specific to the mycobacterial cell wall triggering an exceptionally strong immunogenic response compared to N-acetyl MDP (134). The possibility of a role for NOD2 in non-bacterial infections has also been suggested, with NOD2 having been shown to induce an IFNβ-driven antiviral response following recognition of single-stranded viral RNA (135). Indeed, viral ssRNA from respiratory syncytial virus (RSV), vesicular stomatitis virus (VSV), and influenza virus has been shown to trigger a non-canonical NOD2-directed signaling pathway that requires mitochondrial antiviral signaling protein (MAVS) and induces IRF3 activity, leading to the production of type I IFNs (135) (Figure 4). However, it is still unclear whether lack of NOD2 results in susceptibility to viral infection in humans.

NOD1 and 2 are encoded by the *CARD4* and *CARD15* genes respectively, and as NLRCs, both contain the shared NOD and LRR domains in addition to an amino-terminal CARD. Despite the strong similarities between the two receptors, differences exist; NOD1 contains one CARD domain, while NOD2 contains two (136) and expression of NOD1 is detected in a wide variety of cell types, whereas NOD2 expression is restricted to myeloid cells (136–138), keratinocytes (139) and intestinal, lung, and oral epithelial cells (140–142).

Activation of NOD1 and 2 follows the cytosolic recognition of peptidoglycan ligands that triggers oligomerization of the receptors via their NOD domain and the recruitment of mediators needed to form a signaling complex referred to as the nodosome (143). The nodosome is directed to the point of bacterial entry on the plasma membrane of polarized epithelial cells by the regulatory protein FRMBP2 (144). NOD1 and 2 both interact with RIPK2, via a CARD–CARD homotypic interaction (145–148). This association results in the recruitment of a number of E3 ubiquitin ligases, including TNF receptor-associated factors (TRAFs) (149), cellular inhibitor of apoptosis (cIAP)1 and cIAP2 (150), X-linked inhibitor of apoptosis (XIAP) (151, 152), and ITCH (153). K63-linked ubiquitination of RIPK2 has been established as a means to construct protein scaffolds that transduce downstream signaling. In a step-wise fashion, ubiquitination of RIPK2 leads to activation and recruitment of the TAK1 complex, consisting of TAK1 in association with TAK1-binding protein (TAB)2 and 3. The kinase activity of TAK1 leads to phosphorylation events that activate AP-1 and NF-κB. In parallel to cIAP-induced ubiquitination of RIPK2, XIAP’s enzymatic activity results in the formation of polyubiquitin chains on RIPK2, serving as a platform to engage another E3 ligase complex known as the Linear Ubiquitin Assembly Complex (LUBAC) (152, 154). LUBAC attaches linear ubiquitin chains to the regulatory protein NEMO, allowing for activation of the IKK complex. The kinase activity of IKKβ results in the phosphorylation and degradation of the inhibitor of NF-κB (IκB), allowing for NF-κB dimers to translocate to the nucleus and induce proinflammatory gene expression (155). Besides activating NF-κB, NOD1 and NOD2 have also been shown to activate the p38, JNK, and ERK MAPK pathways (147, 156, 157) and to interact with other NLRs such NLRP1 and NLRP12 (158, 159) (Figure 4).

NOD1 and 2 have been implicated in a number of chronic inflammatory diseases. Mutations and SNPs in *CARD15* in particular, have been linked to a multitude of inflammatory diseases including Crohn’s disease (160, 161), Blau Syndrome (162), asthma (163, 164), atopic eczema (165), atopic dermatitis (105), arthritis (166, 167), and sarcoidosis (168). In the context of Crohn’s disease, the most common mutation that confers susceptibility is a frameshift mutation in the LRR region of the receptor (160), while the mutations conferring susceptibility to Blau syndrome occur in the NOD region (162). While the contribution of these mutations to disease is unknown, further work on understanding NOD2 function could unveil the link between the gene and the disease, as well as allow for the creation of new therapies for these chronic and often devastating diseases. Several NOD2 loss-of-function mouse lines have been generated in an attempt to elucidate its role in Crohn’s disease. Pauleau and Murray generated the first *NOD2* knockout mice (156). Surprisingly, these mice lacked symptoms associated with spontaneous intestinal inflammation, although stimulation of primary macrophages from these animals with MDP failed to trigger inflammatory responses, confirming loss of NOD2 activity (156). Other NOD2 mouse mutants were later generated to express common Crohn’s disease susceptibility mutations (157, 169). While these mice did not develop any gut inflammation resembling that of Crohn’s disease patients, they did display increased susceptibility to bacterial infection, and were shown to produce decreased amounts of β-defensins when challenged with *L. monocytogenes* (157, 170). Similarly, mice deficient in NOD1, NOD2, or RIPK2 also exhibited enhanced susceptibility to bacteria including *Helicobacter pylori* (171–173), *Chlamydophila pneumoniae* (174), *L. pneumophila* (172, 175, 176), and *B. anthracis* (177). This susceptibility often resulted from an inability to control bacterial burden, possibly due to a reduced ability to recruit neutrophils as well as a decrease in the production of proinflammatory and antimicrobial molecules (176, 177). Despite the prevalence of NOD2-deficient models, there remains controversy as to whether Crohn’s disease-linked mutations in NOD2 diminish or enhance its activity in the context of the disease. Common Crohn’s disease-associated NOD2 variants expressed in HEK293T kidney cells are unable to detect MDP and activate NF-κB (178) and monocytes from Crohn’s disease patients with the *1007fs* variant displayed defects in the secretion of TNFα, IL-6, IL-8, and IL-10 (179, 180) and many of these NOD2 variants seem to act recessively (181). While these findings point to a loss-of-function effect of the mutations, Karin and colleagues have argued that the models used in these studies lack resemblance to the natural course of the disease in humans and that the Crohn’s disease-associated NOD2 mutations may in fact result in a gain-of-function. Indeed, Crohn’s disease has been associated with the presence of activated NF-κB and inflammatory NF-κB target gene products in epithelial cells and lamina propria macrophages (182, 183) rather than in circulating blood monocytes used in studies with cultured cells, and results from experiments using tissue samples have differed from those using monocytes (179).

The study of NOD2 linkage to Crohn’s disease has been extended to encompass a key role of NOD1 and 2 in the regulation of autophagy. Autophagy is a housekeeping process in which organelles or other cellular components are degraded and recycled into nutrients during times of starvation or stress. This process results in the formation of a double membrane vacuole known as the autophagosome, which fuses with lysosomes to degrade its contents (184). The role of NOD1 and 2 in this process was initially proposed following GWAS findings of an association between a key component of the autophagy process, ATG16L1, and susceptibility to Crohn’s disease (185, 186). Not only have NOD1 and 2 been shown to interact with ATG16L1 (187), but murine Paneth cells expressing the ATG16L1 mutation associated with Crohn’s disease were unable to produce antimicrobial peptides despite NOD2 stimulation (188). Additionally, the autophagic machinery is involved in loading antigen onto MHC Class II, a process that has been observed to be defective in Crohn’s disease (189). Recently, Lupfer et al. further substantiated the link between NOD2 and autophagy by demonstrating a role for NOD2-RIPK2 signaling in the regulation of the NLRP3 inflammasome following infection with influenza A virus. By triggering the phosphorylation of the autophagy inducer ULK1, RIPK2 induces autophagy of disrupted mitochondria (mitophagy), preventing the accumulation of ROS and NLRP3 inflammasome activation. Mice lacking Nod2, Ripk2, or Ulk1 were hypersusceptible to influenza A infection due to a hyperactive NLRP3 inflammasome and excessive IL-18 levels (190) (Figure 4). Collectively, these studies provide evidence for a key role of NOD2 in autophagy-associated processes such as xenophagy, antigen presentation, antimicrobial peptide secretion, and mitophagy.

Other diseases have also been associated with genetic variants in loci encompassing the genes encoding NOD1 and/or 2. GWAS have linked SNPs in *CARD15* to prostate (191) and endometrial (192) cancer, as well as to gastric lymphoma induced by *H. pylori* infection (173). Similarly, SNPs in *CARD15* were linked to susceptibility to leprosy (193, 194) and tuberculosis (195, 196). The observation that *CARD15*, *RIPK2*, and *NF-*κ*B* have been linked to leprosy (193), tuberculosis (195, 197, 198), and IBD (150, 199) by GWAS and other genetic studies in humans and mice, has led to speculation of a common etiology between mycobacterial diseases and Crohn’s disease (197, 200, 201).

### NLRP10

NLRP10 was discovered based on its homology to NLRP3 and APAF1 (202). Lack of LRRs in NLRP10 may indicate a role for this protein as a signaling adaptor rather than an NLR sensor. NLRP10 has been found in human and murine skin (203), colon, kidney, and testis (204), with mRNA and protein expressed in epithelial cells (202, 205) and hematopoietic cells (206). NLRP10 was previously proposed as a negative regulator of NF-κB, cell death, and IL-1β release (202). These results have been supported by NLRP10 over-expression studies in *Nlrp10* knock-in mice and in *in vitro* studies. In the murine model, *Nlrp10* knock-in mice were found to be resistant to LPS-induced endotoxic shock, due to a decreased release of inflammatory cytokines (203). This was consistent with the observation that cells from these animals secreted reduced amounts of IL-1β following infection with *Salmonella* or TLR7 stimulation (203). However, another group proposed a role for NLRP10 in augmenting the NOD1 immune response to *Shigella flexneri*, indicating the possibility of an inflammasome-independent function for NLRP10 (205). While this mechanism is still poorly understood, the ability of NLRP10 to interact with NOD1 as well as its signaling targets RIPK2, TAK1, and NEMO, suggests that NLRP10 may be involved in optimizing cytokine release following bacterial infections. Furthermore, Flavell and colleagues reported a role of NLRP10 in adaptive immunity. Using NLRP10 knockout mice, this group examined T-cell responses to ovalbumin and aluminum hydroxide, complete Freund’s adjuvant with myelin oligodendrocyte glycoprotein, and LPS. Interestingly, *Nlrp10*^−/−^ mice displayed major defects in TH2, TH17, and TH1 responses, potentially due to a defect in the ability of dendritic cells to transport antigen to draining lymph nodes (207). These findings, as well as those of another group that reported hematopoietic compartment-dependent susceptibility of *Nlrp10*^−/−^ mice to *C. albicans* (206), highlight a role for NLRP10 in bridging innate and adaptive immunity. Despite these findings, understanding the role of NLRP10 in immunity is still in its infancy, and applications of this protein to human diseases are limited while the function of NLRP10 remains largely uncharacterized. However, GWAS have linked *NLRP10* to atopic dermatitis (105, 165, 208), an interesting find considering the abundant expression of NLRP10 in the skin.

### NLRX1

NLRX1 is unique among NLRs in that it contains an N-terminal mitochondrial targeting sequence (209, 210). The protein is broadly expressed in the mitochondria, although it is yet unclear whether it is localized to the matrix or to the outer membrane (209, 211). NLRX1 has been shown to enhance ROS production when it is overexpressed (212), following *Chlamydia* (213) and *Shigella* infection, as well as in response to TNFα and poly(I:C) (212). Like NOD2, NLRX1 has been implicated in host antiviral responses following viral RNA detection (135, 212) and has been shown to directly bind both single and double stranded viral RNA via its LRRs (210). Moore and colleagues initially characterized NLRX1 as a negative regulator of MAVS and antiviral signaling (211). Recently, Lei et al. demonstrated a role for NLRX1 and the mitochondrial protein TUFM in enhancing autophagy and reducing type I IFNs following viral infection (214, 215). However, these findings have been contested and the generation of NLRX1-deficient and *Nlrx1* knockdown mice by several groups has produced conflicting results. In some laboratories, the NLRX1 mutant mice did not display any differences in MAVS antiviral signaling compared to wild-type controls (216–219). In contrast, another group’s findings supported their original claim of a role for NLRX1 in inhibiting MAVS signaling pathways (216, 218). Zhao et al. recently associated a missense mutation in the LRR of NLRX1 with susceptibility to chronic hepatitis B infection in human patients. The replacement of the highly conserved Arg707 with a cysteine between a α helix and a β strand was hypothesized to interfere with the electrostatic potential of the region and consequently modulate the activity of the protein (220). Lastly, a group characterizing the molecular signature of SIV-induced gastrointestinal dysfunction found an increase in NLRX1 expression in rhesus macaques 90 days following SIV infection (221). These findings highlight the role of NLRX1 in antiviral defense, but more research is needed to elucidate the precise mechanism.

### NLRC5

One of the newest additions to the NLR family, NLRC5 has been shown to have a similar structure to other NLRs, although the CARD domain has been found to be structurally distinct from CARD domains expressed in other NLRs. The protein is most similar to CIITA, both in structure and activity. NLRC5 has been shown to be able to enter the nucleus, and its main function is believed to be as a MHC Class I transactivator, forming the basis of an enhanceosome for MHC Class I transcription (222). Accordingly, NLRC5 is expressed constitutively in both humans and mice, unlike the more restricted expression of CIITA (223, 224) and knockdown of NLRC5 in cells using siRNA and in knockout mice resulted in a decrease in MHC Class I expression, without significantly affecting expression of MHC Class II (222, 225, 226). Despite the widespread expression of NLRC5, however, there is a distinct upregulation of NLRC5 in lymphocytes compared to other hematopoietic and somatic cells (227, 228). As a key regulator of MHC Class I transcription, NLRC5 expression has been shown to be induced by a number of signals, including IFNβ, poly(I:C), VSV, and LPS (222– 225, 227, 228). However, the most efficient activator of NLRC5 known is IFNγ, which several of the aforementioned signals are known to induce. IFNγ functions via signal transducer and activator of transcription 1 (STAT1) and cannot induce NLRC5 expression in the absence of STAT1 (225, 228).

The effect of NLRC5 on human health and disease has yet to be extensively studied. However, inferences can be made based on NLRC5’s role in MHC Class I presentation and phenotypes observed in NLRC5-deficient mice. Yao et al. observed extreme immunodeficiency in these mice, with the animals unable to mount an effective CD8+ T-cell response when challenged with *L. monocytogenes*. Interestingly, NLRC5 deficiency also seemed to result in a decrease in NLRP3 inflammasome activation, suggesting that NLRC5 may play a role in the regulation of this pathway (226). Murine and cellular models of NLRC5 deficiency have also implicated NLRC5 in the negative regulation of TLR signaling (223, 229, 230), as well as in RIG-I-like receptor signaling(229). However, other groups have disputed these findings (222, 230), and more research needs to be done in order to gain a more comprehensive understanding of the functions of NLRC5.

### Class II transactivator (CIITA)

MHC CIITA was discovered in 1993 as the genetic basis of hereditary major histocompatibility complex Class II deficiency, or bare lymphocyte syndrome (BLS), a disease characterized by severe immunodeficiency due to a lack of MHC Class II expression (231). Its detection via complementation cloning marked the discovery of the first NLR family member, although the classification of NLRs was only later introduced, following the discovery of NOD1. Although CIITA retains the tripartite structure consistent across the NLR family, it contains an additional acidic domain and a proline/serine/threonine (PST)-rich domain at its N-terminus. Unlike other NLRs, the function of CIITA lies in transcriptional regulation of MHC Class II. The previously mentioned additions to the structure of CIITA do not allow it to bind DNA, but provide a platform for the recruitment and interaction of proteins required for the transcription of MHC Class II in leukocytes or other cells following IFNγ stimulation (232–234). Accordingly, CIITA contains nuclear localization signals and nuclear export signals (235–237). As its role suggests, CIITA is expressed in cells that express MHC Class II, mainly lymphocytes, dendritic cells, macrophages, and other professional antigen presenting cells.

In addition to BLS, CIITA has been linked to a number of other human diseases. GWAS and patient exome sequencing studies have linked SNPs in CIITA to celiac disease (238, 239), susceptibility to myocardial infarction (240), rheumatoid arthritis (240–242), multiple sclerosis (240, 242), primary adrenal insufficiency (243), SLE (244), and type 1 diabetes (245, 246), although these results have not always been replicated in subsequent studies (247–249). Gyllenberg et al. suggested that age-dependent variation in the gene encoding CIITA could be responsible for false associations in GWAS (250). Interestingly, women over 75 years of age expressing the rs3087456(G) allele were found to have a higher average bone mineral density and a decrease in bone fractures compared to controls, although the association was not observed in women aged 25 years (251). Ulrich Streidl and colleagues recently used RNA sequencing to identify a novel and frequently expressed *CIITA-BX648577* gene fusion product in the KM-H2 Hodgkin lymphoma cell-line which was associated with decreased HLA Class II expression and increased programed cell death 1 (PDL1) on the surface of affected cells (252). Genomic *CIITA* breaks were found to occur frequently in B-cell lymphoma patients; 38% of primary mediastinal B-cell lymphoma patients and 15% of classical Hodgkin’s lymphoma patients displayed them. The group also observed a decrease in survival in B-cell lymphoma patients expressing genomic *CIITA* breaks compared to control. The role of *CIITA* gene fusion products in B-cell lymphomas remains a field of considerable interest. Understanding the effect of these genomic breaks could lead to novel therapies for a highly treatment-evasive cancer. At the very least, the discovery of these abnormal gene products could lead to the discovery of new biomarkers, which aid clinicians in stratifying patients according to prognosis and predicted therapeutic response.

## Conclusion

NOD-like receptors have been described as master regulators of innate immunity, and research performed on the functions and signaling pathways of these proteins continues to support this claim. NLRs are essential in recognition of microbial- and pathogen-associated molecular patterns (MAMPs and PAMPs), and have the ability to initiate and support robust immune responses through the formation of inflammasomes and the activation of NF-κB, IRF, and MAPK pathways. Functions such as the enhancement of MHC transcription and presentation implicate NLRs in adaptive immunity, and their roles in reproduction, indicate a broader responsibility of this gene family than previously suspected. The potency of NLRs in inducing immune defenses is vital for the host, but can also provide serious problems when dysregulation or malfunction occurs. GWAS have found many SNPs in NLR genes associated with a plethora of inflammatory and autoimmune pathologies. Research is vastly expanding contemporary knowledge on the functions and roles of NLRs, but several NLRs still remain poorly characterized and understood. Specifically, it remains unclear how NLRs can interact with various and structurally diverse ligands. It is hypothesized that upstream receptors or effectors dictate the activation of NLRs. Alternatively, NLRs might employ co-receptors or dimerize with additional sensors to achieve their functions. Further description of the roles of NLRs in initiating and perpetuating human disease, as well as the role of NLRs at the steady state, will prove vital to gaining a comprehensive understanding of many human pathologies and will provide novel targets and therapies for patients afflicted with these diseases.

## Conflict of Interest Statement

The authors declare that the research was conducted in the absence of any commercial or financial relationships that could be construed as a potential conflict of interest.
